# Vitamin-K Antagonists vs. Direct Oral Anticoagulants on Severity of Upper Gastrointestinal Bleeding: A Retrospective Analysis of Italian and UK Data

**DOI:** 10.3390/jcm11216382

**Published:** 2022-10-28

**Authors:** Mattia Brigida, Simona Di Caro, Carmine Petruzziello, Angela Saviano, Maria Elena Riccioni, Francesco Franceschi, Veronica Ojetti

**Affiliations:** 1 Università Cattolica del Sacro Cuore, 00168 Rome, Italy; 2 University College London Hospital (UCLH) NHS Foundation Trust, London NW1 2BU, UK; 3 University College London, London NW1 2BU, UK; 4 Emergency Department, Ospedale San Carlo di Nancy, 00165 Rome, Italy; 5 Endoscopy Unit, Fondazione Policlinico Universitario A. Gemelli IRCCS, 00168 Rome, Italy; 6 Emergency Department, Fondazione Policlinico Universitario A. Gemelli IRCCS, 00168 Rome, Italy

**Keywords:** upper gastrointestinal bleeding, digestive bleeding, severity, anticoagulants, warfarin, vitamin-K antagonist, direct oral anticoagulants, DOAC, emergency department

## Abstract

**Background**: Gastrointestinal bleeding (GIB) is one of most frequent and significant challenges for emergency physicians and gastroenterologists. Mortality for upper (U) GIB is high, especially in the elderly and comorbid patients. However, there is scant evidence in the literature concerning an assessment of warfarin (VKA) and direct oral anticoagulants (DOACs) in terms of upper gastrointestinal bleeding (UGIB) severity. **Aims**: Using data from two different settings (Italy and the UK), we aimed to compare the impact of VKA and DOACs on the severity of UGIB. **Methods**: Retrospective bicentric study on adult patients under VKA or DOACs admitted either to the emergency department at the Gemelli Hospital in Rome, Italy or University College Hospital in London, UK, with suspected UGIB from 01/01/2017 to 31/12/2018. Univariate analysis with Fisher’s exact test, and analysis of variance (ANOVA) were used. **Results**: 106 patients (62 M/44 F; mean age 71.2 ± 16.9 yrs) were enrolled and divided into the VKA group (*N* = 57; M: 56%, mean age: 64.9 ± 21.3 yrs) and the DOAC group (*N* = 49; M: 61%; mean age: 77.6 ± 12.5 yrs). At univariate analysis, the VKA group presented two endoscopic diagnoses more frequently than the DOAC group (26% vs. 8%, *p* < 0.05), were more frequently endoscopically treated (44% vs. 22%, *p* < 0.05), rescoped (12% vs. 2%, *p* = 0.048) and hospitalized (79% vs. 53%, *p* = 0.01) with a longer length of stay, LOS (VKA: 58% > 5 days vs. DOAC: 68% < 5 days, *p* = 0.01). There was no difference in terms of hemoglobin level on admission, however the requirement of blood transfusions was higher in the VKA group (60% vs. 41%, *p* = 0.041). One third of the VKA group showed a lower platelet count than the DOAC group (33% vs. 8%, *p* = 0.01). No statistically significant differences for in-hospital mortality were observed. For the ANOVA, the type of anticoagulant used was the only significant predictor of need to rescope (*p* = 0.041) and a significant co-predictor for a LOS > 5 days (*p* = 0.009; as well as cirrhosis, *p* = 0.013 and age, *p* = 0.005). **Conclusions**: Our outcomes revealed a more severe UGIB in patients on VKA, but the impact of comorbidities (i.e., more cirrhotic patients in the VKA group) cannot be disregarded. DOAC subgroup descriptive analysis, even though on a little cohort, showed higher bleeding severity for rivaroxaban.

## 1. Introduction

Gastrointestinal (GI) bleeding is one of most frequent and complex challenges for the emergency physicians and gastroenterologists, resulting in a considerable number of hospital admissions [1]. The main challenge to face in the emergency department (ED) is to quickly stratify patients based on risk, which is based on literature data and probabilities, therefore consisting of identifying the problem, recognizing life-threatening situations and carrying out appropriate diagnostic and therapeutic strategies in order to improve the patient’s clinical condition. Gastrointestinal bleeding (GIB) requires fast clinical reasoning, diagnostic workup, and different management pathways according to the diagnosis. The lack of detection of a clinically severe condition can lead to a series of complications and, in the worst cases, to a fatal outcome for the patient with GIB [2,3]. 

In particular, epidemiological studies suggest that incidence of upper gastrointestinal bleeding (UGIB) in Europe is variable, with the highest reported incidence in Scotland 172 cases/100,000 inhabitants [4] and the lowest in Denmark, Sweden and the Netherlands 37–48/100,000 inhabitants [5,6]. Yet, the epidemiology of GIB seems to be changing over time and the hospitalization rate is now similar for upper and lower GIB, probably due to a decreased incidence in the former [7]. However, lower (L) GIB tends to have a higher mortality rate, a longer hospitalization and a higher resource utilization compared to upper GIB [8].

Mortality in UGIB is high, especially in the elderly and comorbid patients, with it being reported as 3.5% in nonvariceal bleeding and 15% in variceal bleeding [9,10]. Furthermore, healthcare costs for UGIB management are substantial: the average costs for hospitalization of patients older than 60 years of age are reported as about $5500 per patient in the United States [11], with an annual initial in-hospital treatment for all UGIB cases in the UK of around £155.5 million [12]. 

The sources of UGIB can be divided mainly according to the aetiology (variceal and non-variceal) or the GI tract involved. The most common causes are: (1) in the oesophagus: erosive oesophagitis, oesophageal varices, Mallory–Weiss tear; (2) in the stomach: peptic ulcer, erosive gastritis, congestive gastropathy, gastric varices or neoplasia; (3) in the duodenum: peptic ulcer and erosive duodenitis; (4) finally, Dieulafoy ulcer and angiodysplasia can be found in different portions of the GI tract [13,14,15,16]. 

The endoscopic treatment has revolutionized the management of this condition over the last decades, and it now plays a major role both in diagnosis and treatment [17]. Methods of endoscopic hemostasis for acute UGIB and LGIB include injection (usually diluted epinephrine or a special sclerosing agent), contact and non-contact thermal devices (unipolar or bipolar electrocoagulation, heater probes, and argon plasma coagulation), and mechanical devices (endoscopic clips and band ligation) [18].

In a systematic review published in 2014 involving 2888 patients affected by peptic ulcers, hemoclips alone or injection therapy combined with thermal therapy were more effective than injection therapy alone. Thus, it was concluded that epinephrine injection therapy should not be used as a monotherapy but in conjunction with a secondary therapy in order to achieve a greater success rate in bleeding control [19,20]. 

As is known, medications have a considerable impact on the risk and severity of GI bleeding [21]. On one side, the increasing use of proton pump inhibitors (PPIs) in the general population has played a protective role against peptic ulcer disease [22,23]. On the other side, non-steroidal anti-inflammatory drugs (NSAIDs) and Helicobacter pylori (*H. pylori*) infection represent an important and common risk factor for GI bleeding [24,25].

Furthermore, life expectancy has increased over the past decades, and consequently so has the cohort of comorbidities of the elderly [26]. Diseases such as atrial fibrillation, deep vein thrombosis, pulmonary embolism and patients presenting increased thromboembolic risk require anticoagulation and antiplatelet therapy, [27,28,29,30] exposing them to increased hemorrhagic risk [31,32].

Vitamin K antagonists (VKA) were first used in clinical practice in the second half of the twentieth century in order to reduce the stroke risk in patients with prosthetic heart valves or atrial fibrillation, but also in the management of pulmonary embolism and deep vein thrombosis. It has been estimated that in Western countries, 2% of the population, nowadays, are under treatment with an oral anticoagulant. However, this drug has some drawbacks: frequent international normalized ratio (INR) monitoring and relative dose adjustments in order to maintain it in the correct therapeutic interval, food and drug interactions. Therefore, in the new millennium the more manageable direct oral anticoagulants (DOACs) were introduced: dabigatran (2009), an oral prodrug, converted to the active dabigatran by plasma esterases and that directly inhibits thrombin (factor IIa of coagulation); apixaban (2011), rivaroxaban (2011) and edoxaban (2013) inhibit the coagulation factor Xa. For these drugs there are fewer drug interactions, there is no need for lab monitoring of coagulation status and, consequently, they do not require dose adjustments. During treatment with an oral anticoagulation, the most frequent adverse event is bleeding, and particularly frequent is GIB [33,34,35,36,37].

A meta-analysis conducted about 10 years ago analyzed data from 43 randomized controlled trials (on treatment of venous thrombosis or acute coronary syndrome for a total of 151,578 patients) that compared DOACs with warfarin for risk of bleeding. Their results showed that patients discordances can be found in the literature and new data would be needed to provide an update to the experiences of the past decade [38].

For all the above considerations, it is crucial to establish the impact of DOACs compared to VKA on gastrointestinal bleeding risk in real world experience. Therefore, we aimed to assess and compare patients on VKA or DOACs presenting with UGIB in two different settings, in Italy and in the United Kingdom (UK), in order to highlight differences in terms of their impact on severity of bleeding.

## 2. Methods

### 2.1. Study Design

This is a retrospective, observational, bicentric study conducted at Fondazione Policlinico Universitario A. Gemelli IRCCS, Rome, Italy and University College London Hospital (UCLH), London, UK.

### 2.2. Study Population

All adult (>18 years of age) patients (pts) on either VKA or DOACs, presenting to the ED with hematemesis, melena, syncope and referred anemia at Fondazione Policlinico Universitario A. Gemelli IRCCS or University College London Hospital from 1 January 2017 to 31 December 2018 were eligible for inclusion. All patients must have undergone upper GI endoscopy during ED admission or ward hospitalization. 

Patients presenting to the ED with signs of UGIB secondary to a trauma, foreign body or caustic ingestion were excluded. 

### 2.3. Bleeding Severity

Bleeding severity was established according to: one or more sources of bleeding and relative endoscopic treatment, UGIB recurrence, need for blood transfusions, management in the brief observation unit of emergency department vs. wards, a length of stay (LOS) > 5 days and mortality.

### 2.4. Statistical Analysis

In the descriptive part of the overall population (Gemelli + UCLH) categorical variables (i.e., gender, previous bleeding, UGIB recurrence, comorbidities, clinical presentation, medications, biochemistry, endoscopic diagnosis and treatment, need for blood transfusions, department of hospitalization and LOS) were expressed as relative and/or absolute frequencies, as well as percentage values.

DOAC subgroup analysis was also presented and considering that the specific categories of DOACs were underpowered when taken singularly to allow for inferential statistics, these data were reported as descriptive statistics, with relative and absolute frequencies.

The analytical part was carried out with SPSS 22 software (SPSS Inc., Chicago, IL, USA). The categorical variables were tested with Fisher’s exact test. *p* values were generated and considered at 95% confidence interval (*p* < 0.05) in order to assess statistical significance. Furthermore, to increase the statistical power of the analysis, we conducted a statistical regression with Analysis of Variance (ANOVA) and we considered a significant *p* < 0.05 for the *F*-test.

## 3. Results

### 3.1. Demographics

Overall, 106 patients (62/106 M, 58.5%; 44/106 F, 41.5%) were retrospectively analyzed for the study period (1 January 2017 to 31 December 2018). Mean age was 70.8 ± 15.3 years.

The VKA group was composed of 57 patients (32/57 M, 56.1%; 25/57 F, 43.9%): 28 were from GEMELLI (49.1%) and 29 from UCLH (50.9%). Mean age was 64.9 ± 21.3 years. 

The DOAC group was composed of 49 patients (30/49 M, 61.3%; 19/49 F, 38.7%): 24 were from GEMELLI (49.0%) and 25 from UCLH (51.0%). Mean age was 77.6 ± 12.5 years. 

Results are summarized in Table 1.

### 3.2. Previous Bleeding

No statistically significant difference was found between the two groups in terms of history of a bleeding event: 13/49 patients, 26.5% in the DOAC group vs. 21/57 pts, 36.8% in the VKA group; neither in terms of clinical presentation at ED (hematemesis: VKA 36.8% vs. DOAC 22.5%; melena: 56.1% vs. 63.3%; syncope: 19.3% vs. 12.2%, more than one of these factors 14.0% vs. 10.2%). However, patients in the DOAC group were more frequently referred to the ED for anemia by the general practitioner (GP) compared to VKA (40.8% vs. 21.1%, *p* < 0.05). Results are summarized in Table 1.

### 3.3. Comorbidities and Past Medication History

Comorbidities representing indirect causes of bleeding either for their pathophysiological mechanisms or their related therapy were taken into account.

Interestingly, frequency of alcoholism (*p* < 0.01) was higher in the VKA group compared to the DOAC group (15/57 pts, 26.3% vs. 2/49 pts, 4%, *p* < 0.01); as well as cirrhosis (16/57 pts, 28.1% vs. 2/49 pts, 4.1%—*p* < 0.01) and for mechanical heart valve replacement (11/57 pts, 19.3% vs. 1/49 2.0%—*p* < 0.01). 

Patients under DOACs (24/49, 49%) were more protected from PPIs with respect to VKA (15/57, 26.3%—*p* < 0.05). At the same time, DOAC patients (11/49, 22.6%) were more frequently under antiplatelet therapy compared to VKA (4/57, 7%—*p* > 0.05). There was no statistically significant difference in terms of concomitant therapy with NSAIDs and corticosteroids. Results are summarized in Table 1. 

### 3.4. Clinical Presentation

There was no statistically significant difference regarding presentation of melena, hematemesis, syncope or more than one of these. However, it occurred more frequently that DOAC patients (20/49, 40.8%) were referred to the ED by their GP after lab evidence of anemia, compared to VKA (12/57, 21.1%—*p* < 0.05). Results are summarized in Table 1.

### 3.5. Biochemistry and Blood Transfusion Requirement

There was no statistically significant difference between the two groups in regard to hemoglobin level at admission <7 g/dL (VKA: 13 pts, 22.8% vs. DOAC: 10 pts, 20.4%). Nevertheless, blood transfusion requirement was statistically different in favor of DOACs (VKA: 34/57, 59.7% vs. DOAC: 20/49, 40.1%, *p* < 0.05).

One third of patients in the VKA group showed a platelet (PLT) count <150 × 10^9^/L, (19/57 pts, 33.3%) while in the DOAC one only 4/49 pts, 8.2% showed a reduced PLT counts (*p* < 0.01). 

Specifically, in the VKA group, the INR mostly ranged between 1.5–2.5 at admission (26/44, 59.1%) and normalized at 72 h (32/44, 72.7%). Results are summarized in Table 1 and Table 2.

### 3.6. Upper GI Endoscopy

There was no statistically significant difference in terms of bleeding source identification at oesophagogastroduodenoscopy (OGDS) in the two groups (44 pts, 77.2% vs. 30 pts, 61.2% in the VKA and DOAC groups, respectively). However, in the VKA patients (15/57, 26.3%, *p* < 0.05) two endoscopic sources of bleeding were identified compared to DOAC patients (4/49, 8.2%, *p* < 0.05). Particularly, in the VKA group the most frequent diagnoses were oesophageal varices/congestive gastropathy (2/15, 13.3%). 

Also, VKA patients were more frequently endoscopically treated than DOAC patients (25/57, 43.9% vs. 11/49, 22.5%; *p* < 0.05). The most frequent endoscopic treatments in the VKA group were injective therapy with a sclerosing agent (13/25, 36.0%), followed by clips (10/25, 28.0%) and ligation (7/25, 28.0%). On the other hand, the most frequent endoscopic treatments in the DOAC group were thermal (i.e., argon plasma coagulation; 5/10, 50.0%) and endo-clips application (5/10, 50.0%), followed by injective therapy (3/10, 30.0%).

Among patients with a bleeding source identified, there was no difference concerning the need to apply more than one endoscopic type of treatment to reach hemostasis in the two groups (VKA 27.2% vs. DOAC 13.3%). 

**Figure 1:**
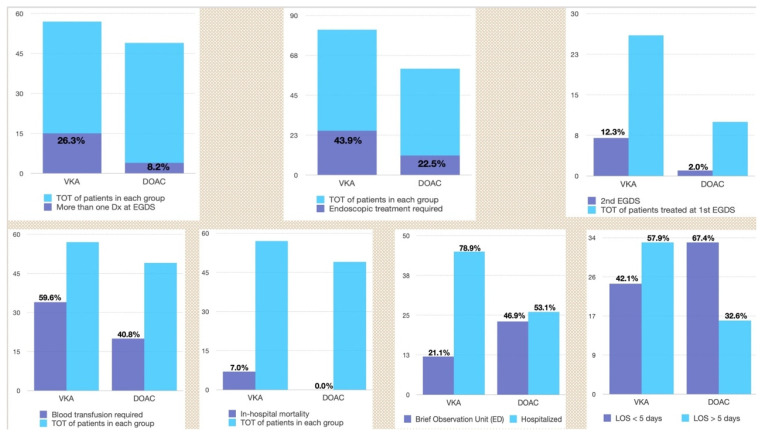
Outcomes of comparison of VKA vs. DOAC in severity of upper gastrointestinal bleeding: more than one diagnosis (top, left); need of endoscopic treatment (top, center); need to rescope (top, right); blood transfusion requirement (down, first graph); management in Brief Observation Unit (Emergency Department, down, second graph) or hospitalization (down, third graph); Length-of-stay of more than five days (down, fourth graph). List of abbreviations: Total (TOT); Vitamin-K antagonists (VKA); Direct oral anticoagulants (DOAC); Diagnosis (Dx); oesofagogastroduodenoscopy (EGDS); Emergency Department (ED); Length-of-stay (LOS).

Among those patients with a bleeding source treated at the first endoscopy, only 1/10 patients in the DOAC group required a second OGDS (10%) compared to 7 patients in the VKA (26.9%) (*p* = 0.05) for UGIB recurrence. Results are summarized in Table 2 (*as “Need to rescope”*) and Figure 1.

### 3.7. In-Hospital Mortality

Although 4 patients died in the VKA group (7.0%) and no patient died in the DOAC group, this result is not statistically significant. In particular, concerning the reported in-hospital deaths in the group, endoscopy showed a gastric ulcer in two of these patients, duodenal ulcer in one patient and finally the fourth patient had negative OGDS whereas a bleeding neoplastic source was subsequently found in the lower GI. Results are summarized in Table 2.

### 3.8. ED Management vs. Ward and Length-of-Stay

Concerning management of these patients, a significantly higher percentage of the VKA patients (79.0%, 45/57 pts) required hospitalization in a ward compared to the DOAC group (53.1%, 26/49 pts): almost half of them could be managed in the brief observation unit of the ED (*p* = 0.01).

Moreover, a statistically significant difference (*p* < 0.01) was found between the two groups in terms of LOS: while more than half of patients belonging to the VKA group (33/57 pts, 57.9%) required hospitalization for more than five days, two thirds of patients in the DOAC group (33/49 pts, 67.4%) required hospitalization of less than five days (*p* = 0.01). 

Results are summarized in Table 2 and Figure 1.

### 3.9. Regression Analysis with ANOVA

Among age, cirrhosis, treatment with antiplatelet therapy, PPIs and VKA/DOACs, all expected as variables influencing the bleeding event, ANOVA showed that the type of anticoagulant used was the only significant predictor of the need to rescope (*p* = 0.041; *F*-test *p* < 0.05) and a significant co-predictor for a LOS > 5 days (*p* = 0.009; as well as cirrhosis, *p* = 0.013 and age, *p* = 0.005; *F*-test *p* < 0.05). 

## 4. DOAC Subgroups Descriptive Data

Out of 49 subjects under treatment with DOACs, 21 patients were under rivaroxaban (42.9%), 15 pts under apixaban (30.6%), 8 pts under dabigatran (16.3%) and 5 pts under edoxaban (10.2%). The full description of the following results (i.e., the outcomes considered for bleeding severity) is reported in Table 3.

Among the different types of DOACs, patients under rivaroxaban (the most used DOAC) had a higher frequency of Hb < 7 g/dL (6/21, 28.6%), of in-hospital UGIB recurrence (1/21, 4.8% vs. no recurrence for the other DOACs) and of hospitalization rate (15/21, 71.4%).

Apixaban patients, instead, showed in only one case Hb < 7 g/dL on admission (6.7%), despite being those with the highest rate of >1 bleeding source found on OGDS (3/15, 20.0%).

Edoxaban patients were those with the highest frequency of >1 endoscopic treatment need (2/5, 40.0%) and more frequently had a LOS > 5 days (3/5, 60.0%).

Finally, dabigatran patients had the highest blood transfusion requirement (2/5, 40.0%).

In-hospital mortality did not occur for any of the DOACs.

## 5. Discussion

Atrial fibrillation, deep vein thrombosis, pulmonary embolism and many other conditions with an increased thromboembolic risk require anticoagulation, usually with an oral anticoagulant (VKA or DOAC), with a consequent risk of GI bleeding as an adverse event [33,38]. However, less data are present in the literature concerning a comparison of bleeding severity between these two categories of anticoagulant medications, especially when it comes to UGIB. The idea of our bicentric research project stems from this gap. Indeed, our study was aimed at retrospectively analyzing and comparing two groups of patients, the VKA group and the DOAC group, from two different tertiary referral centers (Gemelli Hospital, Rome, Italy and University College of London Hospital, London, United Kingdom), in order to highlight differences in terms of anticoagulant impact on the severity of UGIB. Bleeding severity was established according to: one or more sources of bleeding and relative endoscopic treatment, UGIB recurrence, need for blood transfusions, management in brief observation units vs. wards, a length of stay (LOS) > 5 days and mortality. No selection for the center to which patients belonged was applied, so as to increase the statistical power of the analysis. Furthermore, taking into account the latest evidence in the literature on how the COVID-19 pandemic affected trends in ED admissions for a wide variety of acute conditions [39,40,41,42], our idea was to analyze pre-pandemic data, thus we considered a timespan from 1 January 2017 to 31 December 2018.

The population was homogeneous in terms of number and sex, while the mean age was higher in the DOAC group. A small percentage of patients from both groups had episodes of GI bleeding prior to admission, without a statistically significant difference. However, considering the retrospective nature of the study and the only partial data concerning the exact time when anticoagulant treatment was prescribed for each patient, it was not possible to draw conclusions regarding how frequent a bleeding event can occur during anticoagulant exposure over time in either group.

Taking into account the comorbidities in the two groups, with a reasonable explanation we noted that mechanical heart valve replacement, history of alcoholism and cirrhosis (the latter two per-se related to each other) were mainly present in the VKA patients. The reason for this distribution has to be attributed to the main indication to use heparin or warfarin, while less data are present concerning DOAC use, both for mechanical heart valve replacement and for portal vein thrombosis, a known complication of cirrhosis-related portal hypertension [43,44,45,46]. No statistically significant differences came to light between the two groups concerning the other comorbidities considered for our purposes.

For both groups, we considered other concomitant medications that might further increase the bleeding risk (antiplatelet agents, NSAIDs) or reduce it (PPIs): DOAC patients were at the same time more protected by PPIs and more exposed to antiplatelet agents than VKA patients, while the frequency of NSAIDs use was equally distributed in the two groups. 

Concerning the clinical presentation, melena, hematemesis, syncope (or more than one of these), they were equally distributed in the two groups. However, we also recorded whether the patient was referred to the ED by the GP after lab evidence of anemia, and we found out that this occurred more frequently for the DOAC patients. This might appear odd, considering that patients under VKA have to undergo frequent INR monitoring [47] and are thus keener to have closer medical attention for dose adjustment. 

On the other hand, regarding blood workup on admission, there was no difference in terms of hemoglobin level < 7 g/dL in the two groups. At the same time, VKA patients more frequently received blood transfusion compared to DOAC patients, leading to a more severe evolution of the clinical picture and a greater fragility of the patients under warfarin. Furthermore, the higher presence of cirrhosis in the VKA group might explain why in the same group thrombocytopenia and increased bilirubin levels on admission seemed more frequent. The presence of an altered kidney function was equally represented. INR levels on admission for the VKA group, whose value is known to increase as the prothrombin time is affected by dicoumarol agents [48], were below the therapeutic interval for most of these patients; in particular, almost half had an INR of 1.5–2.5 and one third had an INR of <1.5. This might be attributed to VKA stop for some of these patients as the bleeding event occurred, to a delayed seeking of medical attention, to food–drug interactions or to an incorrect anticoagulant intake by the patient.

Regarding endoscopy, a more severe picture was found at OGDS in the VKA group: patients from this group usually had more than one source of bleeding endoscopically identified, and more frequently required endoscopic treatment. Among those patients with a bleeding source treated at the first endoscopy, VKA patients more frequently required a second endoscopy compared to DOAC patients.

Furthermore, two important differences were brought to light by comparison of the two groups that strongly contribute to suggest more severe upper GI bleeding in VKA patients. 

First of all, almost 80% of these patients had to be hospitalized, while there was no difference in terms of where the DOAC patients were managed (ED vs. ward). Second, almost 60% of VKA patients had a length of stay of more than five days, while almost 70% of DOAC patients were hospitalized for fewer than five days.

Finally, concerning in-hospital mortality, there was no statistically significant difference between the two groups. 

Overall, VKA patients more commonly had more than one source of bleeding found at endoscopy, more frequently received endoscopic treatment and were rescoped, had a longer LOS and were more often hospitalized compared to the DOAC patients, without a statistically significant difference concerning in-hospital mortality.

Given the scarcity of data in the literature regarding a comparison of UGIB severity in the two cohorts of patients that we studied, we could compare only some of our outcomes with other studies. For example, Brodie et al. [49], in their retrospective analysis on patients with GIB under DOACs, VKA and a control non-anticoagulant group with outcomes and results similar to ours, found that DOAC patients required fewer hospitalizations and fewer blood transfusions, despite greater concomitant antiplatelet use compared to warfarin. Moreover, an observational Danish cohort study using nationwide registries analyzed data of patients admitted for GIB under DOACs (*N* > 2000 pts) and VKA (*N* > 3500 pts) and found no statistically significant difference in mortality in the two groups [50], and this is in line with our results.

Our statistical regression with ANOVA showed that the type of anticoagulant used was the only significant predictor of the need to rescope and a significant co-predictor for a LOS > 5 days (as well as liver cirrhosis and age). 

This result needs to be discussed from two standpoints: on one side, it highlights the burden that comorbidities can exert on the outcomes of a bleeding event; on the other side, applying this to our study, where we had a higher prevalence of liver cirrhosis in the VKA group, it needs to be stressed that a cirrhotic subject is a complex and potentially fragile patient. Thrombocytopenia, a frequent finding in cirrhotics (and more frequent in our VKA cohort), is usually secondary to splenomegaly with hypersplenism as a consequence of portal hypertension [51,52], and the impact of this reduced blood cell line on hemostasis might in part be responsible for a greater need to rescope these patients compared to DOACs. Moreover, cirrhotic changes lead to significant alterations in coagulation homeostasis for a number of reasons [53,54]. Furthermore, the two most frequently associated endoscopic diagnoses that we found in VKA were oesophageal varices and congestive gastropathy, once again two typical findings in cirrhotic patients [55,56]. Indeed, we take into account the caution that must be used when interpreting these results, considering that other authors already stressed the impact that comorbidities might exert on the outcomes of a bleeding event. On this matter, a French prospective multicenter study evaluated patients with UGIB under oral anticoagulants and found that DOACs, when compared to VKA, did not alter their outcomes (rebleeding in the first 6 weeks, need for surgery/interventional radiology, mortality) and they commented that comorbidities are one of the most important factors affecting UGIB prognosis [57]. Similarly, Di Minno and colleagues, in their review, discussed how severity of comorbidities can exert a greater impact on the risk of GIB than the type of antithrombotic agent used [58]. Finally, it must be said that the indirect effect that warfarin has on coagulation status lasts longer compared to the more direct action of DOACs, which is therefore more closely dependent on the drug half-life. Indeed, as well as the fact that reaching a therapeutic INR interval when taking warfarin can take some days, the same is true for an INR normalization after stopping this treatment [59,60,61], thus leading a possible impact of this aspect on the longer length of stay of VKA patients to be considered.

One last comment that should be made is that we analyzed DOACs taken altogether (rivaroxaban, apixaban, edoxaban, dabigatran) due to the relatively small sample of patients retrieved in each DOAC category for the study period, so we could only conduct descriptive statistics for the single DOAC, and we reported it for a matter of completion. Therefore, even though we cannot infer concerning statistically significant difference among the DOACs, we registered the highest relative frequency of hemoglobin < 7 g/dL on admission, a higher relative frequency of UGIB recurrence and a higher relative frequency of hospitalization rate in the rivaroxaban subgroup. 

This study could pave the way to future projects focused on a larger DOAC population, in order to find the best one in terms of GI bleeding risk profile and then compare it to warfarin on a larger and therefore statistically stronger population.

Moreover, since non-variceal and variceal bleeding were included in this study as to increase the statistical power, further studies separating these two subgroups might give even more precise results on the impact of VKA and DOACs. 

Furthermore, many studies can be found in the literature regarding the risk of bleeding in VKA vs. DOACs, but very few have been conducted on the severity of the bleeding event. Therefore, this aspect might be investigated in future studies.

### Limitations

Limitations to our study might have been the lack of characterization of *H. pylori* for those patients with gastric or duodenal peptic ulcers in our analysis, the retrospective nature of the study, and missing data concerning *follow-up*. Furthermore, even though the bicentric nature of the study allowed for a larger sample of patients, the authors are aware of the sometimes different management carried out by the two centers in the two different countries.

## 6. Conclusions

This study was aimed at comparing two arms of patients based on the anticoagulant used (VKA vs. DOACs) from two different tertiary referral centers (Fondazione Policlinico Universitario A. Gemelli IRCCS, Rome, Italy—University College of London Hospital, London, UK) in terms of risk and severity of UGIB.

VKA patients had more than one source of bleeding found at endoscopy, more frequently received endoscopic treatment and were rescoped, had a longer LOS and were managed in a ward rather than ED compared to DOAC patients. Not only do our results confirm that there is a difference in terms of different bleeding risks according to the anticoagulant category, as the literature shows, but they also demonstrate a diverse impact of VKA vs. DOACs on the severity of upper GI bleeding (a matter that is less investigated in the literature). Furthermore, our data corroborate the idea that the impact of comorbidities should always be taken into account when assessing outcomes of a bleeding event, as the presence of cirrhosis in our VKA group is likely to have given a certain contribution to a greater bleeding severity in this cohort.

Plus, our subgroups analysis of the different types of DOACs has shown that patients under apixaban had higher hemoglobin levels on admission compared to the other DOACs; at the same time, we registered the highest relative frequency of hemoglobin < 7 g/dL on admission, a higher relative frequency of UGIB recurrence and a higher relative frequency of hospitalization rates in the rivaroxaban subgroup. However, the DOAC cohort, when divided into subgroups, could only allow for descriptive statistics. Therefore, this study could pave the way to future projects focused on a larger DOAC population, in order to find the best one in terms of upper and lower GI bleeding severity and risk profile.

## Figures and Tables

**Table 1:** Patients’ Characteristics.

	VKA vs.	DOAC	*p*-Value
**Demographics**			
Patients (N)	57	49	
Gender	M: 32, 56.1%	M: 30, 61.3%	0.60
	F: 25, 43.9%	F: 19, 38.7%	0.60
Mean Age (years)	64.9 + 21.3	77.6 + 12.5	
**Home therapy**			
PPIs	15 (26.3%)	24 (49.0%)	0.03
VKA	-	-	
DOAC	-	-	
Antiplatelet	4 (7.0%)	11 (22.6%)	0.03
NSAIDs	5 (8.2%)	4 (8.8%)	1.0
Corticosteroids	3 (4.1%)	2 (5.3%)	1.0
More than 1 (no PPIs)	2 (6.1%)	3 (3.5%)	0.66
**History of previous GI bleed**	21 (36.8%)	13 (26.5%)	0.30
**Comorbidities**			
Diabetes	20 (35.1%)	11 (22.5%)	0.20
Hypertension	27 (47.4%)	28 (57.1%)	0.34
Atrial fibrillation	16 (28.1%)	20 (40.8%)	0.21
Mechanical heart valve	11 (19.3%)	1 (2.0%)	<0.01
Stroke	1 (1.8%)	3 (6.1%)	0.33
TIA	0 (0.0%)	2 (4.1%)	0.21
Pulmonary embolism	3 (5.3%)	6 (12.2%)	0.30
DVT	9 (15.8%)	9 (18.4%)	0.80
History of neoplasia	15 (26.3%)	13 (26.5%)	1.0
Alcoholism	15 (26.3%)	2 (4.1%)	<0.01
Cirrhosis	16 (28.1%)	2 (4.1%)	<0.01
Other hepatopathy	3 (5.3%)	1 (2.0%)	0.62
Autoimmune disease	3 (5.3%)	5 (10.2%)	0.47
Osler–Weber–Rendu disease	2 (3.5%)	0 (0.0%)	0.50
Previous abdominal surgery	4 (7.0%)	3 (6.1%)	1.0
**Clinical presentation**			
Melaena	32 (56.1%)	31 (63.3%)	0.55
Hematemesis	21 (36.8%)	11 (22.5%)	0.14
Syncope	11 (19.3%)	6 (12.2%)	0.43
Referred anemia	12 (21.1%)	20 (40.8%)	0.03
More than one (no referred anemia)	8 (14.0%)	5 (10.2%)	0.77
**Biochemistry**			
**Haemoglobin**			
>12 g/dL	9 (15.8%)	5 (10.2%)	0.57
9.1–12 g/dL	15 (26.3%)	17 (34.7%)	0.40
7–9 g/dL	20 (35.1%)	17 (34.7%)	1.00
<7 g/dL	13 (22.8%)	10 (20.4%)	0.82
**Platelet count**			
150–450 × 109/L	37 (64.9%)	43 (87.8%)	<0.01
<150 × 109/L	19 (33.3%)	4 (8.2%)	<0.01
>450 × 109/L	1 (1.8%)	2 (4.1%)	0.60
**INR**			
<1.49	19 (33.3%)	-	-
1.50–2.50	26 (45.6%)	-	-
2.51–4.00	5 (8.8%)	-	-
>4.00	7 (12.3%)	-	-
**Increased Bilirubin**			
(>1.2 mg/dL)	21 (36.8%)	3 (6.1%)	<0.01
**Increased Creatinine**			
(>1.17 mg/dL)	20 (35.1%)	17 (34.7%)	1.00

List of abbreviations: Vitamin-K antagonists (VKA); direct oral anticoagulants (DOACs); proton pump inhibitors (PPIs); non-steroidal anti-inflammatory drugs (NSAIDs); gastrointestinal (GI); transient ischemic attack (TIA); deep vein thrombosis (DVT); international normalized ratio (INR).

**Table 2:** Upper GI endoscopy, blood transfusion, in-hospital mortality, management setting, length of stay.

	VKA (*N* = 57)	vs. DOAC (*N* = 49)	*p*-Value
**UGI Endoscopy**			
**-Findings:**			
Oesophagitis	6 (10.5%)	2 (4.1%)	0.28
Oesoph. Varices	10 (17.5%)	2 (4.1%)	0.03
Congestive gastropathy	4 (7.0%)	0 (0.0%)	0.12
Erosive gastritis	7 (12.3%)	6 (12.2%)	1.0
Erosive duodenitis	2 (3.5%)	1 (2.0%)	1.0
Gastric ulcer	9 (15.8%)	5 (10.2%)	0.57
Duodenal ulcer	9 (15.8%)	6 (12.2%)	0.78
Other ulcers ^1^	4 (7.0%)	2 (4.1%)	0.68
Other minor diagnoses ^2^	7 (12.3%)	10 (20.4%)	0.30
Negative	13 (22.8%)	19 (38.8%)	0.09
More than one	15 (26.3%)	4 (8.2%)	0.02
**-No. of treatments ^3^**			
None	19 (43.2%)	20 (66.7%)	0.03
One	13 (29.5%)	6 (6.7%)	
Two	10 (22.7%)	4 (13.3%)	0.25
Three	2 (4.5%)	0 (0.0%)	
**-Therapy ^4^**			
Injective	13 (52.0%)	3 (30.0%)	0.46
Thermal	6 (24.0%)	5 (50.0%)	0.22
Clips	10 (40.0%)	5 (50.0%)	0.71
Hemospray	3 (12.0%)	0 (0.0%)	0.55
Ligation	7 (28.0%)	1 (10.0%)	0.40
**-Need to rescope ^4^**			
Once	7 (26.9%)	1 (10.0%)	0.05
Twice	0 (0.0%)	0 (0.0%)	
**Blood transfusion**	34 (59.6%)	20 (40.8%)	0.04
**In-Hospital Mortality**	4 (7.0%)	0 (0.0%)	0.12
**Management setting**			0.01
Brief Observation Unit (ED)	12 (21.0%)	23 (46.9%)	
Ward	45 (79.0%)	26 (53.1%)	
**LOS**			
24–48 h	4 (7.0%)	12 (24,5%)	0.01
48–72 h	7 (12.3%)	7 (14.2%)	0.78
3–5 dd	13 (22.8%)	14 (28.4%)	0.51
>5 dd	33 (57.9%)	16 (32.6%)	0.01

^1^ Other ulcers: oesophageal, Dieulafoy, anastomotic ulcers. ^2^ Other minor diagnosis group includes: polyposis, suspected cancer, Barrett’s oesophagus, Mallory–Weiss tear, gastric varices. ^3^ Out of OGDS + patients (N-VKA = 44; N-DOAC = 30). ^4^ Out of OGDS+ patients who were treated (VKA = 25; DOAC = 10). List of abbreviations: vitamin-K antagonists (VKA); direct oral anticoagulants (DOACs); upper gastrointestinal (UGI); emergency department (ED).

**Table 3:** Descriptive table on DOAC subgroup. Severity of anaemia, upper GI endoscopy, blood transfusion, UGIB recurrence, management setting, length of stay, in-hospital mortality.

	Apixaban	Rivaroxaban	Edoxaban	Dabigatran
**N (%)**	15/49 (30.6%)	21/49 (42.9%)	5/49 (10.2%)	8/49 (16.3%)
**Hb < 7 g/dL on admission**	1/15 (6.7%)	6/21 (28.6%)	1/5 (20.0%)	2/8 (25.0%)
**>1 bleeding source at OGDS**	3/15 (20.0%)	1/21 (4.8%)	0/5 (0.0%)	0/8 (0.0%)
**>1 endoscopic treatment**	1/15 (6.7%)	1/21 (4.8%)	2/5 (40.0%)	0/8 (0.0%)
**Blood transfusion**	5/15 (33.3%)	9/21 (42.9%)	2/5 (40.0%)	5/8 (62.5%)
**UGIB recurrence**	0/15 (0.0%)	1/21 (4.8%)	0/5 (0.0%)	0/8 (0.0%)
**Hospitalized**	8/15 (53.3%)	15/21 (71.4%)	3/5 (60.0%)	3/8 (37.5%)
**LOS > 5 days**	4/15 (26.7%)	7/21 (33.3%)	3/5 (60.0%)	1/8 (12.5%)
**In-Hospital mortality**	0/15 (0.0%)	0/21 (0.0%)	0/5 (0.0%)	0/8 (0.0%)

List of abbreviations: Direct oral anticoagulants (DOACs); Haemoglobin (Hb); oesophagogastroduodenoscopy (OGDS); upper gastrointestinal bleeding (UGIB); length of stay (LOS).
